# Sharper scissors: How TREX2 fine-tunes CRISPR-Cas9 nickases for precise editing

**DOI:** 10.1016/j.omtn.2024.102119

**Published:** 2024-01-19

**Authors:** Marc Güell

**Affiliations:** 1 Universitat Pompeu Fabra, Barcelona, Spain; 2 ICREA, Institució Catalana de Recerca i Estudis Avançats, Barcelona, Spain

The field of genetic engineering has been revolutionized by the advent of CRISPR-Cas9 technology,^1^^,^^2^ a powerful tool for precise genome editing. Very recently, this technology produced the first approved medicine.^3^ A recent study^4^ published in *Molecular Therapy Nucleic Acids* focuses on a novel approach involving TREX2 and paired CRISPR-Cas9 nickases to enhance genome-editing efficiency and safety. This commentary aims to dissect the study’s methodology, results, and implications for future research and applications.

CRISPR-Cas9 technology allows for targeted modifications of the genome by creating double-strand breaks (DSBs) at specific locations in human cells.^5^^,^^6^ Cas9 has two main catalytic domains, RuvC and HNH, which cleave the non-target DNA strand and the target DNA strand, respectively. These catalytic domains work together to create a DSB in the DNA when Cas9 is guided to a specific target site by its associated guide RNA (gRNA). However, this process can sometimes lead to unintended mutations or off-target effects. To mitigate these risks, researchers have been exploring the use of “nickases”—modified CRISPR-Cas9 enzymes that induce single-strand breaks (nicks) rather than DSBs. Inactivation of a single Cas9 catalytic site yields a “nickase,” which only cleaves one of the two strands of the DNA. This approach requires two gRNAs guiding the corresponding nickases to two opposite DNA strands near the same site, significantly reducing off-target effects while still enabling effective gene editing.^7^^,^^8^

Endogenous DNA repair machinery has been a fruitful source of factors for enhancing or modulating CRISPR-based genome editing.^9^ Wang et al.^4^ focus on TREX2, a factor introduced to enhance the efficiency of genome editing mediated by paired CRISPR-Cas9 nickases. TREX2 is known for its 3′ exonuclease activity, which can process DNA ends. TREX2 is shown to expedite repair mechanisms or make the nicks more conducive to edits, especially when the nicks result in 3′ overhanging ends.

The authors compared genome-editing efficiency in cells treated with CRISPR-Cas9 nickases with and without TREX2; they observed important increases of efficiency in 3′ overhanging ends both for SceI nuclease and Cas9 nickases. They did not observe improvements in Cas12a, likely due to 5′ overhangs produced by this nuclease. This study was based on human HEK293T and mouse embryonic stem cell (ESCs). A non-homologous end joining (NHEJ) reporter and a selection of endogenous sites were analyzed. In addition to increased efficiency of on-target editing on 3′ overhangs, the presence of TREX2 promoted full or near-full deletion of the targeted region, increasing the precision of the gene-editing process. The enhancement produced by TREX2 is deletion-size dependent, and it varies among different sites. An NHEJ reporting system and next-generation analysis of edited cells were used to determine the rate and accuracy of the intended genomic modifications. Interestingly, off-target levels of double nicking in presence of TREX2 were not increased.

The authors also designed mechanistic studies to better understand the system. Interestingly, TREX2 overexpression can stimulate editing, but its elimination does not inactivate NHEJ of blunt ends, 3′ overhanging ends, or 5′ overhanging ends.^10^ Loss- and gain-of-function studies involving TREX2 and XRCC4 seem to suggest that endogenous processing of 3′ overhanging ends is not performed by TREX3 but is perhaps permitted by XRCC4, whose inactivation very much reduces NHEJ levels.

The integration of TREX2 with paired CRISPR-Cas9 nickases represents an interesting development in the field of genetic engineering. By potentially increasing the efficiency and precision of CRISPR-mediated genome editing, this approach holds promise for a range of applications. For instance, highly precise CRISPR tools are important for applications where specificity is critical, including allele-specific editing or repetitive genomic region engineering. Another interesting feature is the increase in the percentage of complete deletions, reducing the heterogeneity of the gene-editing outcome. This may be especially interesting in germline-editing applications where chimerism is an important challenge. However, further research is needed to fully understand the mechanisms and long-term implications of this method, including germline applications and *ex vivo* and *in vivo* deployment demonstrations. For instance, it has been shown recently that paired nickases can significantly reduce AAV self-insertion in mouse liver.^11^ As the authors tested the system in human HEK293T and mouse ESCs, it will be very relevant to see how these advances translate into more biotechnological and therapeutically relevant systems.

Overall, the present study is a valuable contribution to the evolving landscape of genetic engineering. It underscores the ongoing efforts to refine CRISPR technology for safer and more effective applications.
